# Recent Advances and Remaining Challenges in the Management of Diabetic Kidney Disease

**DOI:** 10.3390/jcm12082759

**Published:** 2023-04-07

**Authors:** Guillermo Gervasini

**Affiliations:** Department of Medical and Surgical Therapeutics, Medical School, Institute of Molecular Pathology Biomarkers, University of Extremadura, 06005 Badajoz, Spain; ggervasi@unex.es

Diabetic kidney disease (DKD), which refers to pathologic structural and functional changes observed in the kidneys of patients with diabetes mellitus (DM), is the greatest contributor to CKD and the most common cause of end-stage kidney disease (ESKD) worldwide. DKD affects roughly 40% of patients diagnosed with diabetes, contributing significantly to their morbidity and mortality. In addition, and unlike other diabetes complications, DKD prevalence has failed to decline in recent decades, becoming a growing socioeconomic burden. Consequences derived from DKD include decreased glomerular filtration rate, increased albumin levels, proteinuria, fluid retention, elevated arterial blood pressure and renal failure. Furthermore, these DKD patients are at such an elevated cardiovascular (CV) risk that they are far more likely to die from CV disease than from deterioration in their renal function.

In the last two years, several relevant contributions published in the *Journal of Clinical Medicine* (*JCM*) have been made to the field of DKD. These have addressed issues such as treatment options, the need for an early diagnosis, the extremely complex pathophysiology of the disease and the implementation of new biomarkers that can help to identify individuals at risk of faster progression and/or more prone to developing CV events.

The paper by Sawaf and colleagues [1] reviews the current landscape of treatment strategies for DKD. This is an exciting field, because after decades of having angiotensin-converting enzyme inhibitors and angiotensin receptor blockers as the only options for these patients, several very promising alternatives are presently available. The authors successfully describe how, for example, the benefits of sodium-glucose transport protein 2 inhibitors (SGLT2i) have been observed to go beyond their glucosuric effect, as they also display independent renoprotective and CV actions, an ideal scenario for DKD patients. More recently, a number of clinical trials have demonstrated that Glucagon-like peptide-1 receptor agonists (GLP-1ra) have CV and renal benefits as well, paving the way for their use in DKD. Two other useful reviews on each of these drug groups have also been recently published in *JCM* [2,3]. These novel therapies, along with other promising agents, such as dipeptidyl peptidase-4 (DDP-4) inhibitors, may radically change the way we are currently handling DKD. Sawaf et al. also stress the fact that a more personalized approach may be possible by applying tailored treatments based on genetic and biomarker profiles. In this regard, Mota-Zamorano et al. [4] published an interesting study in the journal, highlighting the role that the epoxygenase pathway of arachidonic acid metabolism may play in DKD. In over 1000 subjects, these researchers showed that a V433M variant in the *CYP4F2* gene, which is responsible for the synthesis of 20-HETE, a major mediator in hyperglycemia-induced renal cell damage, was associated with a decreased risk of DKD. In addition, carriers of this variant displayed lower urinary levels of 20-HETE after correcting for renal function, which may explain the protective effect of the 433M allele. These findings add to the existing body of evidence pointing to this metabolic route as a promising therapeutic drug target in DKD.

A pressing problem in DKD is the lack of accurate biomarkers of early diagnosis and prognosis. Albuminuria has traditionally been the hallmark of DKD; however, 20% of DKD patients do not present with albuminuria. In addition, nephropathy can also go unnoticed at the time of diagnosis. These facts highlight the need for novel biomarkers that are able to characterize more precisely these patients’ subgroups. In a very interesting paper, Saenz-Pipaon et al. [5] evaluate the emerging role of extracellular vesicles (EVs) as putative noninvasive alternatives for the early diagnosis and prognosis of DKD. EVs are lipidic nanospheres released by most cell types that carry nucleic acids, metabolites and proteins from the parent cell, thus participating in intercellular communication. It has been suggested that particularly urinary EVs might mirror the structural and functional changes experienced by the kidney in DKD. This review shows that EVs might play a relevant role in DKD development and progression, very likely by mediating fibrosis-related pathways in target cells. Determining the concentrations and specific types of EVs holds the potential to make them useful markers of DKD. It should be noted, however, that the present limitations of the techniques designed to separate and characterize EVs may delay their implementation in routine clinical practice.

In another issue of the journal, a research group from Spain [6] addresses the issue of monitoring DKD progression, which is essential to be slowed down in order to reduce mortality and morbidity in these patients. The risk factors associated with a decline in the glomerular filtration rate (GFR) are described in this review, as well as different ways to measure it, either directly, e.g., by radioisotopes or radiopharmaceuticals, which is a method not implemented in routine clinical practice, or indirectly, by using formulas such as Cockcroft–Gault, MDRD-4 or CKD-EPI, with the latter being the method currently recommended. The authors also emphasize the concept already mentioned in this editorial that albuminuria is quite a late indicator of DKD, as tissue injury is already established by this point. The presence of hyperfiltration before albuminuria being observed should warn the clinician to start CV and renal protective therapy. A second part of this review deals with the major outcomes utilized in clinical trials that evaluate the use of new therapies (SGLT2i or GLP1ra) in DKD. García-Carro et al. argue that the heterogeneity of these measured outcomes makes comparisons difficult, and they debate the pros and cons of standardizing the main CV and renal endpoint definitions. Such standardization, the authors conclude, would make clinical trials more homogenous, thus making meta-analyses easier and facilitating tailored treatments for diabetic patients at risk of ESKD.

The pathogenesis of DKD is multifactorial, which poses a challenge for both basic and clinical research. Knowledge of the mechanisms leading to DKD onset is yet to be fully elucidated, and a variety of biochemical pathways have been involved. In this regard, the levels of fibroblast growth factor (FGF) 23 have been observed to increase with the onset of diabetes, whilst they are also strong predictors of disease progression and mortality in CKD patients. Another group of Spanish researchers [7] analyzed the ongoing debate regarding the existence of a determining role of FGF23 imbalances on the incidence of DM and its complications, e.g., DKD. They also proposed a mechanistic basis for the reported associations. The elevation of FGF23 levels via pathological states would create a maladaptive effect in pancreatic beta cells and immune cells that could translate into decreased insulin production and the stimulation of the synthesis of proinflammatory factors. The authors also stress the importance of paying attention to alpha-Klotho levels, as this protein works in combination with FGF23, which can actually function as an effector of Klotho effects on insulin sensitivity.

A different type of biomarker that may also be advantageous for improving the understanding of the intricate pathophsysiology of DKD is the image-based biomarker. Renal magnetic resonance imaging (MRI) methods have experienced remarkable development in the last decade, as they provide noninvasive information on renal volume, function, metabolism, perfusion, oxygenation and microstructural alterations without the need for exogenous contrast media. In the first issue of June 2021, Mora-Gutiérrez et al. [8] summarized the most promising renal MRI techniques in the assessment of DKD, namely arterial spin labeling (ASL)-MRI, blood-oxygen-level-dependent (BOLD)-MRI and diffusion-weighted and diffusion tensor imaging (DWI/DTI)-MRI. Other techniques such as dynamic nuclear polarization MRI or quantitative magnetization transfer, which have also been used in DKD studies, still require technical validation. A perennial drawback of these MRI techniques is their high cost compared to classic urinary and blood biomarkers; therefore, cost-effective studies need to be carried out to evaluate whether this economic burden would be justified, particularly regarding renal MRI implementation in clinical routine practice.

In summary, the different papers published in *JCM* described herein highlight the current advances in the management of DKD, whose treatment is being revolutionized by new drugs such as SLGT2i and GLP-1ra after decades of stalling. On the other hand, more efforts must be made to improve the knowledge of DKD pathophysiology and, particularly, to find novel biomarkers that can help with the early identification of diabetic patients at risk of renal impairment.
